# Advancing pre-clinical surgical education by using intuitive short videos

**DOI:** 10.1186/s12909-025-06895-4

**Published:** 2025-02-28

**Authors:** Marie Perrin, Markus Schäfer, Pierre-Alexandre Bart, Dieter Hahnloser

**Affiliations:** 1 Division of Visceral Surgery, Centre Hospitalier Universitaire Vaudois (CHUV), Rue du Bugnon 44, Lausanne, CH-1011 Switzerland; 2 Medical School, University of Lausanne, Lausanne, Switzerland

**Keywords:** Educational videos, Surgical education, Pedagogical techniques, Medical students, Digital learning platform

## Abstract

**Background:**

The ever-expanding field of surgery requires novel tools to teach surgical pathologies and their management. Basic knowledge must already be acquired on a pre-graduate level at medical school. The SARS-CoV-19 pandemic has pushed students to explore digital online platforms to complement their medical education. This study aimed to assess the utility of short educational videos and the importance of digital learning methods to teach abdominal surgery.

**Methods:**

A literature review was performed for a thorough understanding of educational videos. Short videos were then created covering different topics of abdominal surgery. To evaluate the utility of such videos, three consecutive cohorts of medical students were compared. The first cohort comprised students studying without the videos (V0), whereas the second and third cohorts had access to the videos (V1, V2). Between the three groups, the general demand for videos, subjective study habits, and objective examination scores were compared. In the V1 and V2 group, satisfaction and engagement regarding the videos were also assessed.

**Results:**

This study included 746 medical students over a three-year period, with similar demographics. The demand for videos was high (90% in V0, 88% in V1 and V2 each) in all three groups. In total, 23 short videos were produced. Students recognized the benefits of videos in understanding the basics of surgical pathologies and their management. On average, 95.5% of the students found that videos were successful in summarizing the lectures’ key points. Moreover, 96.5% found that the videos helped them to better recall the lecture content. A median overall improvement of 12.5% between V0 and V1 examination results was observed.

**Conclusions:**

This study emphasizes the importance of implementing innovative teaching methods in modern medical education. Students expressed a strong demand for short educational videos. In the future, this project could expand to other surgical and non-surgical specialties.

**Supplementary Information:**

The online version contains supplementary material available at 10.1186/s12909-025-06895-4.

## Background

Current medical students, belonging to the generations Y (born 1981–1996) and Z (born 1997 or later), can be described as digital natives [1–3]. They use videos daily for various purposes and learn differently than previous generations [4–8]. Different types of online contents, e.g. prerecorded lectures, animated videos, podcasts, and interactive cases, allows students to acquire new information at their own speed, without any constraints of time or location [1, 9–13]. This has been shown to improve learning in numerous studies [1, 9, 14–16]. Hence, medical educators who often belong to digital immigrant generations, have to learn using innovative teaching methods to motivate and attract the attention of younger students [10, 11, 13, 17–19]. It is important to note that actually five generations of physicians are now working and learning together, an unique situation which has never happened before [20]. 

Videos may transmit information in a very expressive way, resulting in vastly improved learner experiences, knowledge retention, and understanding of the lecture’s content [6, 13, 16]. Furthermore, students consider video materials very engaging [6, 9, 12, 14, 15]. The challenge for medical educators is to use these new technologies effectively to transform learning into a more collaborative and personalized experience [10]. 

This study aimed to assess the demand and utility of educational videos among third-year medical students at the University of Lausanne. Indeed, courses have typically been delivered in a very traditional manner, with ex-cathedra lectures in large auditoriums. However, there has been a strong initiative to modernize teaching methods to improve learning in visceral/abdominal surgery.

## Methods

This prospective study was conducted at the Medical school, University of Lausanne, in association with the Lausanne University Hospital, between October 2020 and January 2023. The study was approved by the dean of the Medical school. Three consecutive third-year medical student classes (V0 = year 2020/2021, V1 = year 2021/2022 and V2 = year 2022/2023) were compared. The third-year’s first semester is composed of three sequential modules, whereby the second module contains abdominal surgery lectures. Following this class-intensive period, four weeks devoid of lectures are dedicated for the preparation of three semestrial examinations, one for each module.

**Fig. 1:**
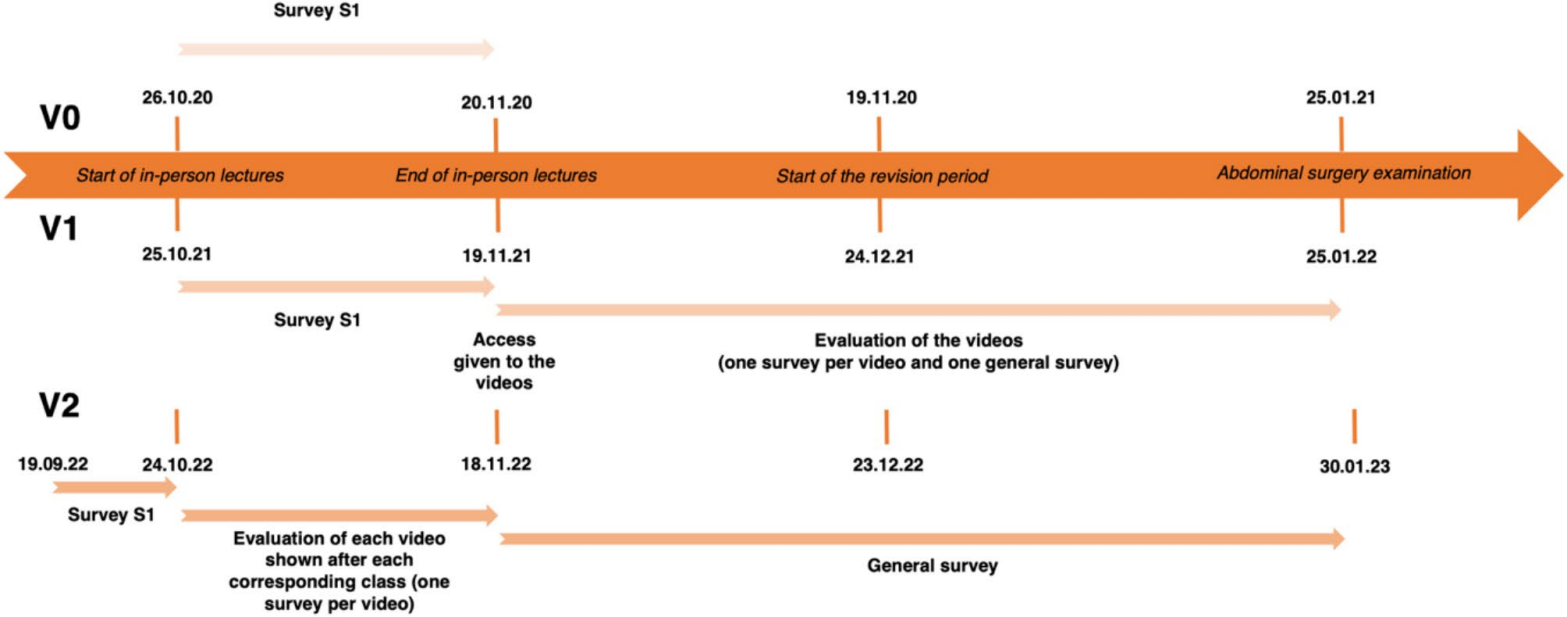
Project timeline

The V0 cohort only received in-person lectures (only available as a live stream due to the SARS-CoV-19 pandemic). The V1 and V2 cohort studied in a blended learning approach using in-person lectures (available face to face as well as a live stream) and the animated educational videos. The V1 group received the videos all at once after having finished all in-person lectures. In comparison, the V2 group received the videos over a 4-week period. Indeed, each video was first shown to the V2 students during the corresponding lecture and was then directly available on the university’s online platform. Figure 1 outlines the timeline for all three cohorts.

### Survey assessing the demand for educational videos (S1)

The same questionnaire was given to all three cohorts prior to having access to the videos. The survey assessed study habits, utility of the educational videos and the students’ preference when the videos should be accessed during the learning process (Appendix 1).

### Literature review and creation of the videos

To create appropriate and efficient videos, a vast literature research was done using PubMed with the following search words: “educational videos”, **“**surgical education”, “pedagogical techniques”, “medical students”, “digital learning platform” and cross reference. After analyzing the literature, short, animated videos for all abdominal surgery lectures were created using Microsoft PowerPoint^®^ (version 16.60) and Adobe Illustrator^®^ (version 2021). Respective lecturers were consulted to guarantee the accuracy of the video’s content.

According to the literature, to maximize the benefits of educational videos, three aspects are vital: motivating students to watch the videos, managing the cognitive load of each video and promoting active learning (Table 1) [14]. 

**Table 1:** Criteria used to create effective videos

Criteria	Examples used in this study
Motivation	• Short video lengths • Examination-relevant information • Modern and enjoyable design • Representational illustrations • High-yield video topics
Cognitive load management	• Videos structured using a similar model • Few rapidly moving visual elements • Highlighting of important information • Uniform introduction
Active learning	• Open-ended questions in each video with time to answer them

### Motivation

The main reason for keeping the videos short, is that student’s engagement is strongly associated with video length [6, 14, 21–23]. Moreover, efforts were made to increase students’ motivation to watch the videos. For example, each video was designed for one respective lecture and contained examination-relevant material [14, 24]. A modern and enjoyable design was also used [14]. It has been shown that the use of visual aids helps students to organize complex contents into meaningful images in their mind, facilitating learning compared to purely text-based methods [25]. Therefore, time was spent on creating representational illustrations in order to serve as the backbone from which students could build their own mental representation of a subject. The chosen topics were frequent and well established in order to minimize the risk yearly modifications [16]. 

### Cognitive load

To make educational videos effective, particular attention was given to managing their cognitive load [6, 14, 26]. For instance, each video followed the same structure and avoided the excessive use of rapidly moving visual elements [21]. Moreover, signals were used to highlight important information [14, 25, 26]. A uniform introduction was created so that students could easily know which video they were viewing [21, 26]. 

### Active learning

In order to promote active learning, each video contained several open-ended questions stimulating self-reflexion [14]. If the time was insufficient to answer these questions, students had the option to pause the video.

Once the videos were available, V1 and V2 students were regularly reminded by email and through the student classes’ WhatsApp^®^ group to watch and evaluate them, as well as during the lectures for the V2 group using a QR-code leading directly to the corresponding survey. All videos were available to students via the university Moodle^®^ platform.

### Video evaluation

Three educational parameters were inquired: (A) satisfaction (surveys, see Appendices 2, 3 and 4), (B) examination performance (multiple choice questions results), and (C) students’ engagement in using the videos (indicators extracted from Moodle^®^ statistics). Videos were evaluated individually as well as after the students had viewed all of them. The global pertinence, appreciation, and utility of this new learning format was assessed by the students and the teachers.

### Statistical analysis

Descriptive statistics included median and range for continuous variables as well as frequencies and percentages for binary and categorical variables. Categorical data were compared by Chi-2 test and continuous variables by T-test. P-values < 0.05 were considered statistically significant.

## Results

### Students

In total, 746 students over three years participated in the study (V0 *n* = 236, V1 *n* = 255, V2 *n* = 255). Students’ demographics were comparable in the three groups, with 68% female in V0, 64% in V1 and 63% in V2.

### Creation of educational videos

By applying the indications found in the literature research, 23 videos were created according to the in-person lectures given on abdominal surgery (See one of the videos in Appendix 5). Video lengths ranged from 1 min 24 s to 5 min 39 s. The average length was 2 min 35 s, and the total duration of all videos was 59 min 13 s (Table 2).

**Table 2:** Length, visualizations, and topic of each video (listed by order of appearance on Moodle)

Video topic	Duration	Total number of views		Number of different viewers (percent)		Number of views per viewer	
		V1*	V2**	V1*	V2**	V1*	V2**
Anal abscess and fistula	1 min 25 s	353	545	192 (75.5)	204 (80)	1.8	2.7
Appendicitis	1 min 24 s	300	632	186 (72.9)	206 (80.8)	1.6	3.1
*Bariatric surgery*	1 min 53 s	265	439	172 (67.5)	198 (77.7)	1.5	2.2
Post-operative complications	3 min 17 s	253	490	160 (62.8)	201 (78.8)	1.6	2.4
Diverticulosis and diverticulitis	2 min 59 s	260	544	161 (63.1)	201 (78.8)	1.6	2.7
Abdominal examination	3 min 23 s	176	262	130 (50.9)	146 (57.3)	1.4	1.8
Hemorrhoids and anal fissures	2 min 45 s	210	418	147 (57.7)	192 (75.3)	1.4	2.2
Inguinal hernia	2 min 56 s	207	413	145 (56.9)	190 (74.5)	1.4	2.2
Hernias	2 min 30 s	196	421	140 (54.9)	192 (75.3)	1.4	2.2
Ileus	2 min 50 s	235	507	146 (57.3)	199 (78)	1.6	2.6
Mesenteric ischemia	2 min 10 s	211	443	134 (52.6)	193 (75.7)	1.6	2.3
Benign lesions of the liver	4 min 02 s	198	452	127 (49.4)	192 (75.3)	1.6	2.4
Crohn’s disease	1 min 25 s	188	401	127 (49.4)	179 (70.2)	1.5	2.2
Acute pancreatitis	2 min 25 s	209	494	131 (51.4)	191 (74.9)	1.6	2.6
Chronic pancreatitis	1 min 51 s	188	419	123 (48.2)	184 (72.2)	1.5	2.3
Biliary tract diseases	5 min 39 s	215	558	130 (51)	195 (76.5)	1.7	2.9
Ulcerative colitis	1 min 29 s	184	350	127 (49.4)	174 (68.2)	1.5	2.0
Lower gastrointestinal bleeding	2 min 34 s	192	383	127 (49.4)	175 (68.6)	1.5	2.2
Splenomegaly and spleen trauma	2 min 52 s	182	453	122 (47.8)	177 (69.4)	1.5	2.6
*Solid organ transplantation*	2 min 42 s	172	201	123 (48.2)	114 (44.7)	1.4	1.8
Abdominal trauma	2 min 28 s	163	342	125 (49)	170 (66.7)	1.3	2.0
Pancreatic tumors	2 min 54 s	194	435	127 (49.4)	182 (71.4)	1.5	2.4
Peptic ulcer and perforation	1 min 20 s	196	445	131 (51.4)	188 (73.7)	1.5	2.4
**Mean**	2 min 35 s	215,1	436,8	140,6 (55.1)	184,5 (72.4)	1,5	2,4
**Median**	2 min 34 s	198	439	131 (51.4)	191 (74.9)	1,5	2,3

Note that the videos in *italic* were not shown during the class for the V2 group

*Numbers collected during a 67-day viewing period, from initial access to examination date

** Numbers collected during a 98-day viewing period, from initial access (following each respective class on a 4-week period) to examination date

### Students request video-based learning

Response rate to the survey S1 between the three cohorts significantly differed though the absolute differences were small (V0 = 90.3%, V1 = 87.8%, V2 = 78.4%, *p* < 0.001). In the three cohorts, only 7 to 14% (V0 = 14/213, V1 = 16/224, V2 = 27/203) of students reported studying topics before attending the respective lectures. However, if a short educational video would be available, the majority would have watched it to prepare for the lecture, to review the lecture and to prepare for the examination (Appendix 6). In all cohorts, the overall demand for educational videos was high.

Most students reported using the lecture’s PowerPoint slides (V0 = 94%, V1 = 94%, V2 = 91%) and their personal notes taken during the lecture (V0 = 84%, V1 = 87% and V2 = 75%) to review the lectures and prepare for the examination. Only 20 to 33% used internet videos, including resources such as YouTube^®^, Osmosis^®^ or Khan Academy^®^. In the three cohorts, less frequently used resources included text or table summaries, internet platforms such as Wikipedia^®^ and Amboss^®^, and published scientific books (details can be found in Appendices 7a, b and c).

**Fig. 2:**
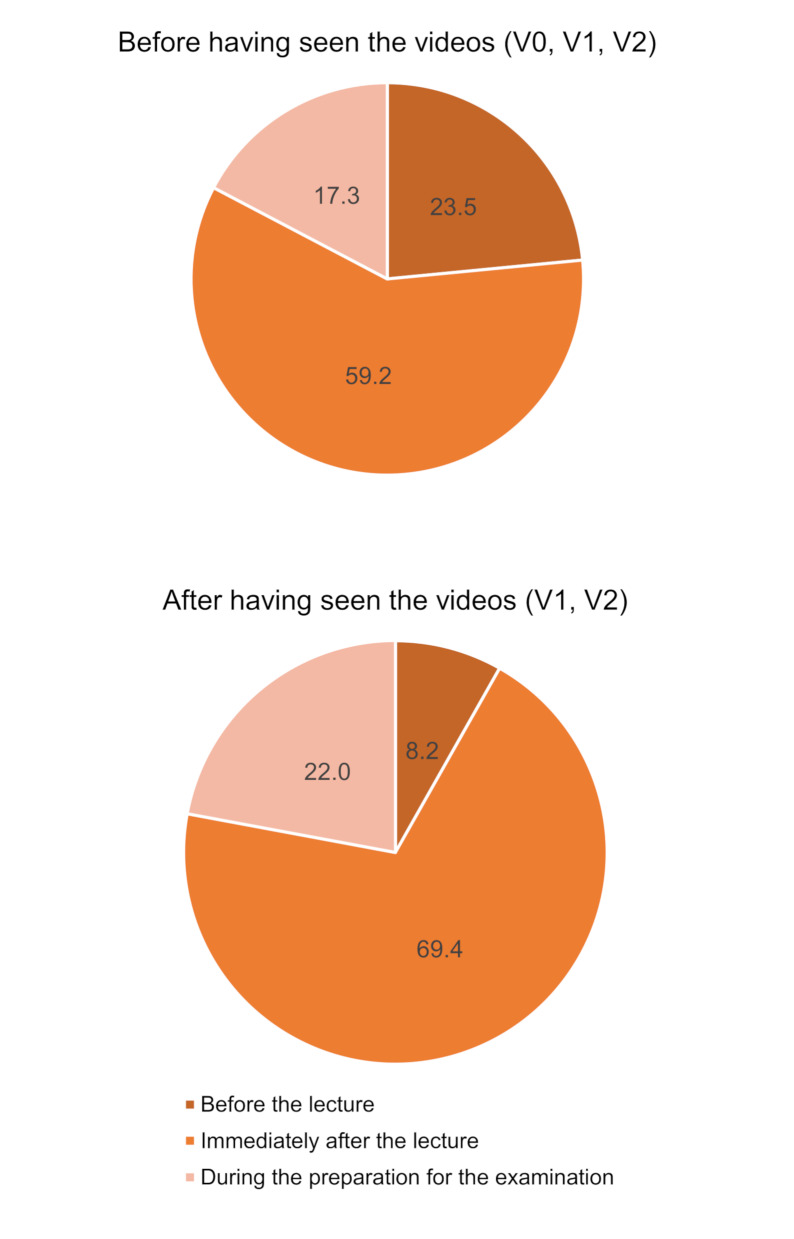
Time points at which the students would prefer to watch the videos. 3**a**: before having access to the videos (V0, V1, V2); 3**b**: after having actually watched the videos

Prior to introducing the videos to any group, students in all cohorts said they would prefer access to videos immediately after the lecture (60% in V0, 62% in V1 and 56% in V2, *p* = 0.016) compared to before the lecture or during the examination preparation. This remained the case in V1 and V2 after having viewed the videos (59% and 80%, respectively, *p* = 0.002, Fig. 2), with V2 responding significantly higher to viewing the videos immediately after the lectures.

### Videos are an incredibly effective tool to improve learning

#### Satisfaction

The videos helped 86% and 95% (V1 and V2, respectively, *p* = 0.017) of students in their learning of abdominal surgery and 89% and 96% (V1 and V2, respectively, *p* = 0.026) to remember the lectures more easily. In the V1 and V2 group, 98.9% and 100% (*p* = 0.835) of students would recommend viewing these videos as part of the abdominal surgery teaching module, respectively.

The response rate to each individual video was low (V1 = 24%, range: 19–40%; V2 = 18%, range: 3–62%, Appendix 8). However, though the following results were not statistically significant, a mean of 94% and 97% (V1 and V2, respectively, *p* = 0.108) of students found that the videos were successful in summarizing the key points of the lecture. Similarly, a mean of 95% and 98% (V1 and V2, respectively, *p* = 0.099) of students found that the amount of information presented in the videos was appropriate and that the videos helped to better recall the content presented during the corresponding in-person lecture (V1 = 96% and V2 = 97%, *p* = 0.085).

The five lecturers were satisfied with the content, the amount of information and the design of the videos corresponding to their classes. Moreover, in 96% of the cases, they found that the corresponding video complemented the lecture well. According to them, the best moment to give the students access to their videos is during or after the lecture (80%), in comparison to during the examination preparation (20%) or before the lecture (0%).

#### Examination performance

The MCQ examination, presented every year to the students, contained 18 (2021, V0 group), 22 (2022, V1 group) and 19 (2023, V2 group) questions on abdominal surgery topics. Between V0 and V1, 10 questions remained identical, whereas V2 had different questions. Regarding the 10 identical questions, V1 increased the median overall score by 12.5% (*p* = 0.225, Table 3). Overall, the median percentage of correct answers for all abdominal surgery questions was V0 = 67%, V1 = 87% and V2 = 60%.

**Table 3:** Examination question subjects and percentages of correct answers in V0 and V1 groups

Question number and subject	Percentage of correct answers in V0 (*n* = 232)	Percentage of correct answers in V1 (*n* = 251)
1 – Acute abdomen	31.9	32.3
2 – Abdominal pain	79.7	69.3
3 – Anal bleeding	70.7	86.5
4 – Diverticulitis	68.1	92
5 – Acute abdomen	84.9	98
6 – Abdominal trauma	99.1	99.6
7 – Proctology	65.9	53.8
8 – Abdominal pain	77.2	87.3
9 – Hepatobiliary surgery	91.4	93.2
10 – Hepatobiliary surgery	71.6	82.5
**Median of correct answers**	74.4	86.9

#### Students’ engagement in using the videos

##### Audience retention

Over a 67-day period, the videos accumulating the highest number of views and number of different viewers in the V1 group were “Anal abscess and fistula” (353 views, 192 viewers), followed by “Appendicitis” (300 views, 186 viewers) and “Bariatric surgery” (265 views, 172 viewers). The V2 students accessed each video after the respective lecture, increasing the viewing period up to 98 days. The videos “Appendicitis” (632 views), “Biliary tract diseases” (558 views) and “Anal abscess and fistula” (545 views) accumulated the highest number of views. In both groups, topics focusing on less specific subjects such as “Abdominal trauma”, “Solid organ transplantation” and “Abdominal examination” were among the less consulted videos (Table 2).

##### Key moments of the audience

**Fig. 3:**
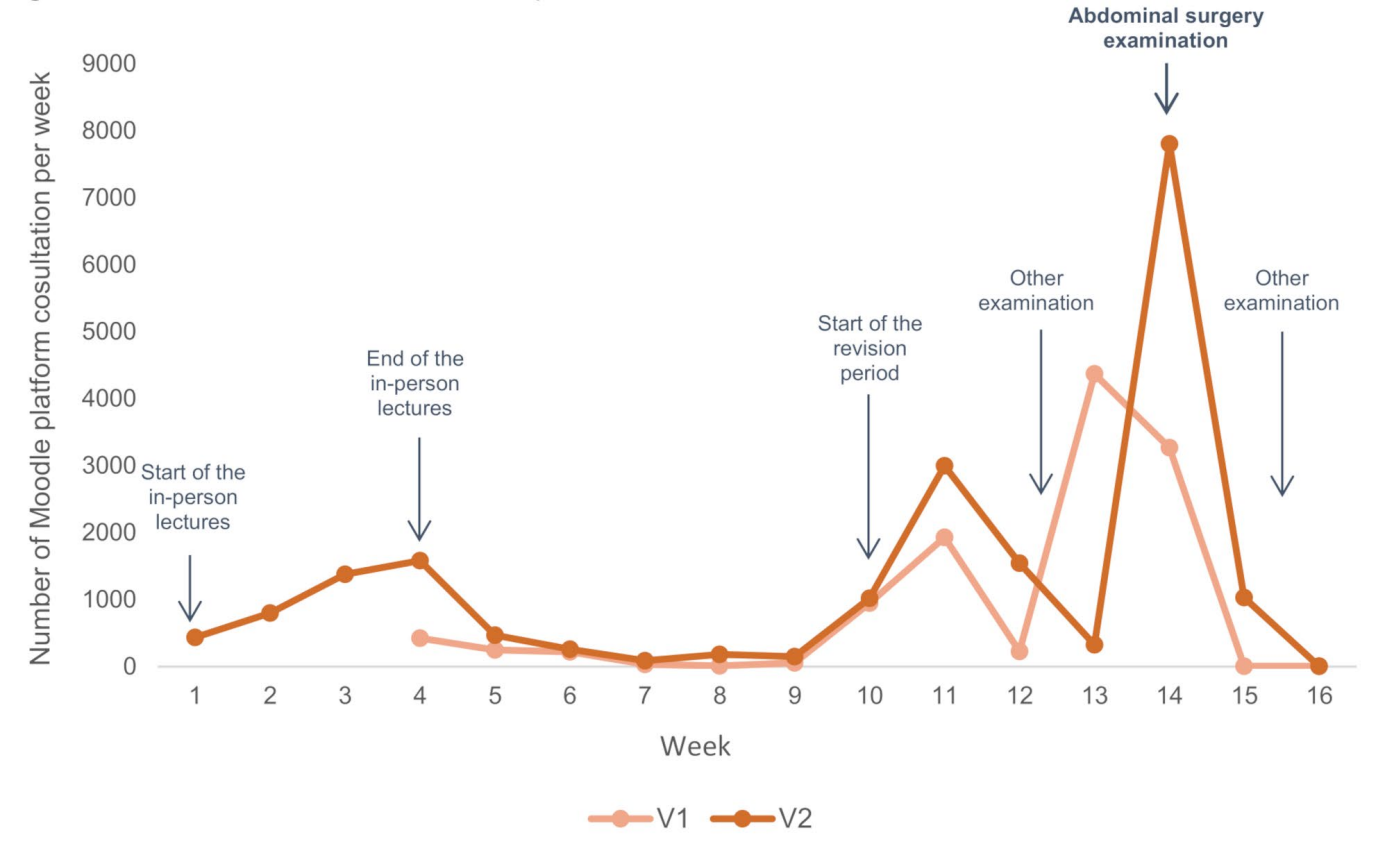
Number of Moodle consultations per week while videos were available

In both groups, videos were most often consulted at two key moments during the study period: the start of the examination preparation phase and just before the abdominal surgery examination (Fig. 3). Furthermore, viewing the videos immediately after each corresponding lecture (V2 group) increased the audience (4371 to 7808 Moodle platform consultations per week) and the number of times a video was watched (1.5 to 2.4 times per video). The length of the videos did not impact the number of views.

## Discussion

Educational videos that accompany surgery lectures were considered to be very helpful by third-year medical students, especially if they were immediately available after the lectures. These videos were well-received tools for summarizing lecture content, improving knowledge retention, and supporting preparation for examination. This highlights the potential of integrating video-based learning into traditional teaching methods to meet the student needs.

The traditional concept of surgical lecture is now being challenged and questioned by emerging novel teaching methods [9, 27, 28]. and demands of a new generation of students [17, 18, 27, 29, 30]. One of the major differences between generations Y and Z compared to previous generations is their relationship with technology [2, 10, 31]. This offers an opportunity for medical educators to assess the application, sustainability and relevance of new learning techniques in their classrooms [31–34], as we did in this study. Indeed, the use of digital tools in combination with in-person lectures, known as the “blended learning” technique, can improve the quality of medical education [1, 6, 9, 19, 30, 35]. Educational videos will have a prominent role in modern medical education, starring in many blended and online lectures [13–16, 18, 21]. Well-designed videos may be used by educators, ultimately reducing the time needed to create high-quality educational content [11, 14, 16, 36]. The novelty of our study is that we applied this new teaching format to abdominal surgery.

The paradigm of 21st -century learning is shifting from an educator-centric to a learner-centric model [13]. From the point of view of the medical students, traditional learning needs the implementation of novel tools, e.g. educational videos, and teachers are increasingly forced to become familiar with such novel technologies to provide high quality lectures. However, for many academic educators, teaching is just one task among many others. An increased administrative load, more involvement in research and pressure to be clinically productive has led to a higher demand on academic faculty members. This has resulted in less time being spent on teaching [1, 9, 37]. The SARS-CoV-2 pandemic has further forced medical schools to quickly shift from in-person to E-learning teaching methods.

Nowadays, medical students wish to have customized learning materials, accessible from anywhere, at any time. The students in this study mainly used the PowerPoint slides and their own notes for revision of the lectures, while scientific books and internet videos were rarely used. This showed that students prioritize using resources directly linked to the lecture they attended that is related to several reasons. First, the examination questions at Lausanne’s medical school are created by the teacher who gives the respective lecture. Second, students only have four weeks to prepare for their examinations, and they may not have time using resources not directly linked to the lecture. Even if internet videos and other resources may be much better adapted to their learning styles, the risk of spending time studying something not likely to be tested is too high, as shown in other studies [1, 5]. 

Medical school teaching is often influenced by external factors such as the curriculum and examination organization. The use of E-learning platforms could help avoid superficial learning and allow students to deepen their knowledge [38, 39]. In this study, the long-term effects of this intervention on learning were not examined. It would be interesting if students who studied with the videos took a second examination several months after their participation in the study, to examine the long-term effect of studying with the videos compared to without. According to the literature, lower performing students benefit the most from blended learning approaches including educational videos and reported higher satisfaction in their learning process [6, 40]. 

In addition, to the bias brought on by the MCQ examination format, this study has some other limitations. There might be a potential selection bias and results may not be generalizable to other settings. Also, response rate to the final satisfaction survey were low for both groups, most likely because answering these surveys was optional and had no influence on the final grade. However, views of the videos increased from 215 times (V1) to 436 times (V2). Another potential bias why there was no significant increase in correct answers to the MCQ could by the fact that the examination for the V0 cohort took place online, due to the COVID-19 pandemic, while V1 and V2’s examinations were in person. Students taking the examination online at home may have had lower stress levels, consequently resulting in a higher number of correct answers.

Further studies are warranted in which blended-learning lectures are directly compared in parallel to classical in-person lectures, measuring outcome using an objective examination system. This would help advance our understanding of the modern student’s needs and learning styles, as well as their connection with new technologies. Furthermore, a more accurate way to objectively measure the utility of videos is needed. This could be done using an examination specifically created for the study, not only containing MCQs, but also open-ended questions.

## Conclusions

In conclusion, this study supports the evidence that medical students demand novel methods including short, animated videos. Videos should be customized to the individual lecture and should be best shown immediately after the corresponding lecture and made available online thereafter. Students used such videos most frequently within four weeks before the multiple-choice examination. In the future, such videos could also be used to preparing lectures and increasing digital learning experiences. Future research should track long-term retention of knowledge and experimental designs to compare the effectiveness of various types of video content.

## Electronic supplementary material

Below is the link to the electronic supplementary material.

Supplementary Material 1

## Data Availability

All data generated or analyzed during this study are included in this published article and its supplementary information files.

## Declarations

### Abbreviations

MCQ, multiple choice question.

CHUV, centre hospitalier universitaire vaudois.

COVID-19, coronavirus disease 2019.

SARS-CoV-2, severe acute respiratory syndrome coronavirus 2.
