# Supplementary material for: Advancing pre-clinical surgical education by using intuitive short videos
Source: BMC Med Educ. 2025 Feb 28;25:323. doi: 10.1186/s12909-025-06895-4 (PMC11869550; doi:10.1186/s12909-025-06895-4)
Supplement: Supplementary file 1 — Supplementary Material 1 [file 12909_2025_6895_MOESM1_ESM.docx]

**Appendix**

**Appendix 1: Survey S1 – Video demand and study habits in V0 and V1.**

What gender are you? *

- Female
- Male
- I do not wish to answer

Do you prepare for the lecture in advance? *

- Yes
- No

If yes, which tools do you use?

- Text summaries
- Table summaries
- Internet videos (YouTube, Osmosis, Khan Academy, …)
- Lecture’s PowerPoint slides
- Published scientific books
- Internet platforms (Wikipedia, Amboss, …)
- The notes taken during the lecture
- The lecture recording
- Other: …

If a short educational video was available, would you watch it to prepare for the lecture? *

- Yes
- No

Do you review the lecture after it has taken place (from occasionally to always)? *

- Yes
- No

If yes, which tools do you use?

- Text summaries
- Table summaries
- Internet videos (YouTube, Osmosis, Khan Academy, …)
- Lecture’s PowerPoint slides
- Published scientific books
- Internet platforms (Wikipedia, Amboss, …)
- The notes taken during the lecture
- The lecture recording
- Other: …

If a short educational video was available, would you watch it to review the lecture? *

- Yes
- No

If a short educational video was available, would you watch it to prepare for the examination? *

- Yes
- No

Which tools do you use to prepare for the examination?

- Text summaries
- Table summaries
- Internet videos (YouTube, Osmosis, Khan Academy, …)
- Lecture’s PowerPoint slides
- Published scientific books
- Internet platforms (Wikipedia, Amboss, …)
- The notes taken during the lecture
- The lecture recording
- Other: …

A short video summarizing the most important points of each lecture would be useful to my learning. *

- 1 (strongly disagree)
- 2
- 3
- 4
- 5 (strongly agree)

At what point in your learning process would access to the first viewing of a video summarizing the essentials of the lecture be most useful to you (only one answer possible)? *

- Before the lecture
- Immediately after the corresponding lecture
- During the preparation for the examination

*Mandatory questions.

**Appendix 2: Survey following each video.**

The video’s content summarizes the key points of the lecture well. *

- 1 (strongly disagree)
- 2
- 3
- 4
- 5 (strongly agree)

The amount of information presented in the video is appropriate. *

- 1 (strongly disagree)
- 2
- 3
- 4
- 5 (strongly agree)

The video helped me to remember the in-person lecture. *

- 1 (strongly disagree)
- 2
- 3
- 4
- 5 (strongly agree)

*Mandatory questions.

**Appendix 3: Final evaluation survey on video satisfaction.**

Overall, the videos helped me to learn abdominal surgery. *

- 1 (strongly disagree)
- 2
- 3
- 4
- 5 (strongly agree)

Overall, the videos helped me to remember the lectures more easily. *

- 1 (strongly disagree)
- 2
- 3
- 4
- 5 (strongly agree)

Would you recommend viewing these videos as part of the abdominal surgery learning process (only one answer possible)? *

- Yes, before the lecture
- Yes, immediately after the corresponding lecture
- Yes, but only during the preparation for the exam
- No

*Mandatory questions.

**Appendix 4: Lecturers evaluation survey on video satisfaction.**

The video’s content summarizes the key points of the lecture well. *

- 1 (strongly disagree)
- 2
- 3
- 4
- 5 (strongly agree)

The amount of information presented in the video is appropriate. *

- 1 (strongly disagree)
- 2
- 3
- 4
- 5 (strongly agree)

The video complements my lecture well. *

- 1 (strongly disagree)
- 2
- 3
- 4
- 5 (strongly agree)

It is convenient for me to show the video within the time limit of the corresponding lecture. *

- Yes
- No

At what point do you think access to the first viewing of a video summarizing the key points of the lecture would be most useful to the students (only one answer possible)? *

- Before the lecture
- During the lecture (included in the duration of the lecture)
- Immediately after the corresponding lecture
- During the preparation for the exam

*Mandatory questions.

**Appendix 5: Appendicitis video**

On demand (format .mp4), not uploaded directly on the platform due to large file size.

**Appendix 6: Answers to survey S1.**

|  | **V0 (213 answers)** | **V1 (224 answers)** | **V2 (200 answers)** |
| --- | --- | --- | --- |
| Number of female students | 145 (68.1%) | 143 (63.8%) | 126 (63.0%) |
| **Studying after the lecture** |  |  |  |
| I review the lecture after it has taken place (from occasionally to always). | 208 (97.7%) | 211 (94.2%) | 194 (97%) |
| If a short educational video was available, I would watch it to review the lecture. | 201 (94.4%) | 217 (96.9%) | 195 (97.5%) |
| **Examination preparation** |  |  |  |
| If a short educational video was available, I would watch it to prepare for the examination. | 186 (87.3%) | 206 (92%) | 180 (90.0%) |
| **Demand for educational videos** |  |  |  |
| Short videos summarizing the key points of each lecture would be useful to improve my learning. * | 192 (90.1%) | 198 (88.4%) | 176 (88.0%) |

* Represents students’ evaluations corresponding to scores 4 and 5 using the 5-Point Likert scale (agree and strongly agree to the statement).

**Appendix 7: Students study habits.**

**a** – V0 study habits according to S1 answers.

An additional resource (flashcards) was cited once after the lecture and twice during the preparation for the examination.

**b** – V1 study habits according to S1 answers.

An additional resource (flashcards) was cited once before the lecture, eight times after the lecture and eight times during the preparation for the examination. Another additional resource (3D Anatomy App) was cited once after the lecture.

**c** – V2 study habits according to S1 answers.

An additional resource (flashcards) was cited seven times after the lecture and during the preparation for the examination.

**Appendix 8: V1 and V2 answers to the surveys corresponding to each of the 23 videos.**

| **Video topic** | | **Number of students answering the corresponding survey.**  **(max = 255)** | | **The video’s content summarizes the key points of the lecture well. *** | | **The amount of information presented in the video is appropriate. *** | | **The video helped me to remember the in-person lecture. *** | |
| --- | --- | --- | --- | --- | --- | --- | --- | --- | --- |
|  |  | **V1** | **V2** | **V1** | **V2** | **V1** | **V2** | **V1** | **V2** |
| Anal abscess and fistula | 102 (40%) | | 44 (17,3%) | 90 (88.2%) | 44 (100%) | 92 (90.2%) | 43 (97.7%) | 97 (95.1%) | 44 (100%) |
| Appendicitis | 96 (37.7%) | | 103 (40.4%) | 88 (91.7%) | 101 (98.0%) | 91 (94.8%) | 99 (96.1%) | 91 (94.8%) | 100 (97.0%) |
| *Bariatric surgery* | 74 (29%) | | 29 (11.4%) | 42 (56.8%) ** | 10 (34.5%) *** | 48 (64.9%) | 21 (72.4%) | 47 (63.5%) | 18 (62.0%) |
| Post-operative complications | 70 (27.5%) | | 28 (11.0%) | 69 (98.6%) | 28 (100%) | 70 (100%) | 28 (100%) | 70 (100%) | 27 (96.4%) |
| Diverticulosis and diverticulitis | 75 (29.4%) | | 73 (28.6%) | 75 (100%) | 73 (100%) | 74 (98.7%) | 73 (100%) | 75 (100%) | 73 (100%) |
| Abdominal examination | 55 (22.7%) | | 158 (62.0%) | 53 (96.4%) | 156 (98.7) | 55 (100%) | 153 (96.8%) | 54 (98.2%) | 151 (95.6%) |
| Hemorrhoids and anal fissures | 62 (24.3%) | | 82 (32.2%) | 62 (100%) | 81 (98.8%) | 62 (100%) | 80 (97.6%) | 61 (98.4%) | 80 (97.6%) |
| Inguinal hernia | 61 (23.9%) | | 57 (22.4%) | 60 (98.4%) | 57 (100%) | 59 (96.7%) | 57 (100%) | 60 (98.4%) | 57 (100%) |
| Hernias | 62 (24.3%) | | 45 (17.6%) | 60 (96.8%) | 44 (97.8%) | 59 (95.2%) | 45 (100%) | 60 (96.8%) | 44 (97.8%) |
| Ileus | 66 (25.9%) | | 113 (44.3%) | 65 (98.5%) | 112 (99.1%) | 65 (98.5%) | 112 (99.1%) | 66 (100%) | 113 (100%) |
| Mesenteric ischemia | 53 (20.8%) | | 29 (11.4%) | 53 (100%) | 28 (96.6%) | 52 (98.1%) | 28 (96.6%) | 53 (100%) | 29 (100%) |
| *Benign lesions of the liver* | 56 (22.0%) | | 15 (5.9%) | 56 (100%) | 15 (100%) | 56 (100%) | 15 (100%) | 56 (100%) | 14 (93.3%) |
| Crohn’s disease | 56 (22.0%) | | 27 (10.6%) | 54 (96.4%) | 26 (96.3%) | 54 (96.4%) | 25 (92.6%) | 54 (96.4%) | 26 (96.3%) |
| *Acute pancreatitis* | 57 (22.4%) | | 28 (11.0%) | 54 (94.7%) | 28 (100%) | 54 (94.7%) | 28 (100%) | 54 (94.7%) | 28 (100%) |
| *Chronic pancreatitis* | 55 (22.7%) | | 23 (9.0%) | 54 (98.2%) | 23 (100%) | 54 (98.2%) | 23 (100%) | 54 (98.2%) | 22 (95.7%) |
| *Biliary tract diseases* | 53 (20.8%) | | 15 (5.9%) | 51 (96.2%) | 15 (100%) | 50 (94.3%) | 14 (93.3%) | 52 (98.1%) | 15 (100%) |
| Ulcerative colitis | 54 (21.2%) | | 37 (14.5%) | 53 (98.2%) | 36 (97.3%) | 52 (96.3%) | 36 (97.3%) | 49 (90.8%) | 37 (100%) |
| Lower gastrointestinal bleeding | 53 (20.8%) | | 16 (6.3%) | 51 (96.2%) | 14 (87.5%) | 50 (94.3%) | 15 (93.8%) | 51 (96.2%) | 14 (87.5%) |
| *Splenomegaly and spleen trauma* | 49 (19.2%) | | 18 (7.1%) | 48 (98.0%) | 18 (100%) | 49 (100%) | 18 (100%) | 49 (100%) | 16 (88.9%) |
| *Solid organ transplantation* | 49 (19.2%) | | 7 (2.7%) | 45 (91.8%) | 7 (100%) | 46 (93.9%) | 7 (100%) | 48 (98%) | 7 (100%) |
| Abdominal trauma | 55 (22.7%) | | 22 (8.6%) | 54 (98.2%) | 22 (100%) | 54 (98.2%) | 22 (100%) | 53 (96.4%) | 22 (100%) |
| *Pancreatic tumors* | 55 (22.7%) | | 24 (9.4%) | 52 (94.6%) | 24 (100%) | 51 (92.7%) | 24 (100%) | 52 (94.6%) | 23 (95.8%) |
| Peptic ulcer and perforation | 63 (24.7%) | | 75 (29.4%) | 62 (98.4%) | 74 (98.7%) | 61 (96.8%) | 75 (100%) | 62 (98.4%) | 74 (98.7%) |
| **Mean** | 62.2 (24.4%) | | 46.4 (18.2%) | 58.7 (94.4%) | 45 (97.0%) | 59 (94.9%) | 45.3 (97.6%) | 59.5 (95.6%) | 45 (97.0%) |
| **Median** | 56 (22.0%) | | 29 (11.4%) | 54 (96.4%) | 28 (96.6%) | 54 (96.4%) | 28 (96.6%) | 54 (96.4%) | 28 (96.6%) |

Note that the surveys for the videos in *italic* were not shown during the class using the QR-code for the V2 group.

*Represents students’ evaluations corresponding to scores 4 and 5 using Likert scale (agree and strongly agree to the statement).

**Only answer with more 4 scores than 5 using the 5-Point Likert scale. In all other answers, there was always more 5 than 4 score using the 5-Point Likert scale.

***Only answer with more 3 scores than 4 or 5 using the 5-Point Likert scale. In all other answers, there was always more 5 than 4 score using the 5-Point Likert scale.
